# Utility of fiberoptic bronchoscopy in diagnosis of various lung conditions: Our experience at rural medical college

**DOI:** 10.4103/0970-2113.68306

**Published:** 2010

**Authors:** Batau Bhadke, Radha Munje, Jaywant Mahadani, Amar Surjushe, Pradeep Jalgaonkar

**Affiliations:** *Department of Chest and TB, Shri Vasantrao Naik Govt. Medical College, Yavatmal, India*; 1 *Department of Pathology, Shri Vasantrao Naik Govt. Medical College, Yavatmal, India*; 2 *Department of Dermatology, Venereology and Leprology, Shri Vasantrao Naik Govt. Medical College, Yavatmal, India*; 3 *Department of Medicine, Shri Vasantrao Naik Govt. Medical College, Yavatmal, India*

**Keywords:** Fiberoptic bronchoscopy, foreign body, fungal infection, malignancy, tuberculosis

## Abstract

**Aim:**

To evaluate the utility of fiberoptic bronchoscopy in order to find out the etiology in various lung conditions.

**Materials and Methods:**

Fiberoptic bronchoscopy was performed in 120 adult patients who had persistent opacities on chest radiography in the form of collapse, consolidation, hilar mass and cavity with proper antibiotic course of 1 to 3 months. Bronchoscopic aspirates, brushing and biopsy (as and when required) were taken. Patient with known lung cancer, sputum positive pulmonary TB, recent myocardial infarction, allergic diseases and blood dyscrasias were excluded.

**Results:**

Fiberoptic bronchoscopy was diagnostic in 90 (75%) patients. Bacterial pneumonias were found in 32 (26.66%), malignancy in 28(23.33%), pulmonary TB in 20 (16.66%), fungal pneumonia in 6(5%) and foreign bodies in 4(3.33%) patients. In 30(25%) patients no specific diagnosis was made.

**Conclusion:**

We conclude that fiberoptic bronchoscopy was found to be extremely useful in finding specific etiology of various lung diseases.

## INTRODUCTION

Fiberoptic bronchoscopy (FOB) is an important entity in the armamentarium of procedures listed in diagnosis of respiratory problems. It is a universally accepted procedure both in the diagnosis and therapy of various pulmonary disorders. This procedure allows careful inspection of the bronchial tree for endobronchial lesion and foreign body and also helps in recovery of deep respiratory secretions, brushing and biopsy, which is useful in diagnosis of un-common infections, neoplasm and other non infectious causes. FOB not only helps in assessing the disease area but also provides better bacteriological and histological yield thus helping to reach a definite diagnosis. The present study was undertaken to diagnose various lung conditions like lungs malignancy, tuberculosis, bacterial and fungal infections.

## MATERIALS AND METHODS

The present prospective study was carried out on 120 patients from July 2003 to June 2006 at Rural Medical College, Shri Vasantrao Naik Government College and hospital, Yavatmal, Maharashtra. Detailed clinical history, physical examinations and investigations were carried out. Assessment of coagulation profile was done. ECG was done in patients above 35 years of age. All patients were subjected to sputum examination (acid fast bacilli, Gram staining, culture/sensitivity, KOH staining, malignant cells), chest radiography and hematological examination. Selected patients were advised computerized tomography and ultra sonography of thorax. Patients with persistent opacity on chest radiography, in spite of appropriate antibiotic therapy for one to three months, and having persistent symptoms like cough, hemoptysis, chest pain, were subjected for fiberoptic bronchoscopy. Written informed consent of all study patients was taken. After premedication with IM atropine 0.6 mg bronchoscopy was performed with flexible FOB (Pentax adult type) through trans-nasal route and under topical anesthesia (2% xylocaine). Some patients required sedation with injection Midazolam. Oxygenation was monitored during and immediately after procedure with pulse oxymetry and oxygen administered to maintain saturation >90%.

Appropriate samples such as the bronchoscopic aspirate, brushing and biopsy (whenever necessary) were obtained depending on the lesion after thorough evaluation of endobronchial tree. Protected specimen brush was not used due to unavailability in our institute. Samples were subjected to cytology, histopathology and fungal staining. In suspected cases of bacterial *pneumonia*, initially, Gram stain was performed for identification of organism which was confirmed by culture. In all the patients culture co related with Gram staining. In clinically suspected cases of fungal *pneumonia*, sputum for KOH was done. Bronchial aspirate were subjected for KOH staining. Because of lack of facilities fungal culture or identification of fungal species were not done. All the cases of fungal pneumonia were diagnosed on the basis of clinical, radiological presentation and identification of fungal hyphae on KOH staining of bronchial aspirate. Patients with known lung cancer smear positive pulmonary TB, recent myocardial infarctions and blood dyscrasias were excluded.

## RESULTS

**Figure 1:**
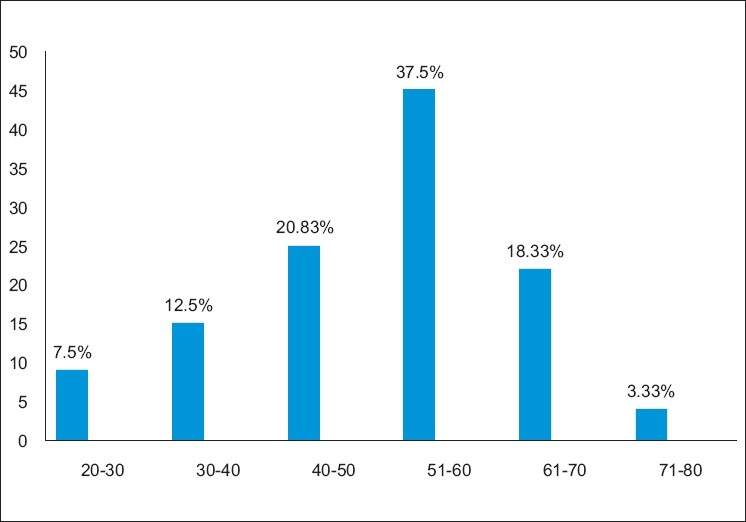
Age distribution of study subjects

The total number of patients enrolled in the study was 120, out of which 80 (66.66%) were males and 40(33.33%) females. The study group belongs to age group of 20–80 years. In the age group 20–30, there were nine (7.5%), in the age group 31–40 there were 15 (12.5%), in the age group 41–50 there were 25 (20.83%), in the age group 51–60 there were 45(37.5%), in the age group of 61–70 there were 22(18.33%), and in the age group of 71–80 there were 4(3.33%) patients [Figure 1]. Highest numbers of patients were found in age group of 51–60 i.e. 37.5%.

**Figure 2:**
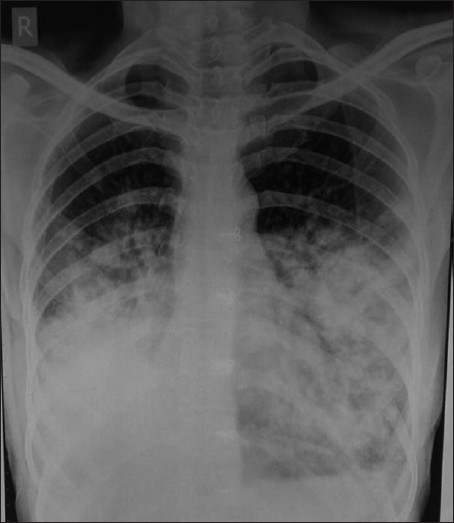
Radiograph showing bilateral lower zone consolidation found to be bronchioloalveolar carcinoma on bronchoscopic aspirate

**Figure 3:**
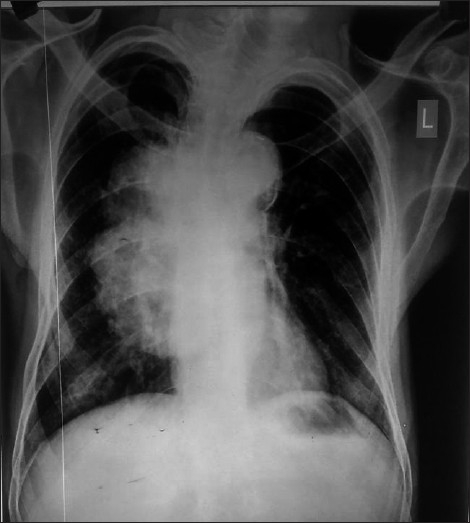
Chest X-ray showing right Hilar mass which was found malignant on bronchoscopy

Out of 120 patients studied, FOB was diagnostic in 90(75%) patients. Bacterial pneumonia were found in 32(35.55%), malignancy in 28(31.11%), tuberculosis in 20 (22.22%), fungal infection in 6 (6.66%) and foreign bodies in 4 (4.44%) cases [Table 1]. In bacterial pneumonia, *Streptococcus pneumoniae* was the commonest organism found in 16 (50%) followed by Staphylococcus in 10 (31.25%) and *Klebsiella* in 6(18.75%) patients [Table 2]. All the patients diagnosed as fungal pneumonia had underlying diabetes mellitus. Radiological examination showed consolidation to be the commonest presentation found in 50 (41.66%) [Figure 2], followed by collapse in 25 (20.83%), parahilar mass in 13 (10.83%) [Figure 3], consolidation with effusion in 8 (6.66%), cavity in 8 (6.66%) and collapse with effusion in 5 (4.16%) patients [Table 3].

**Table 1:** Various etiological factors for non resolving pneumonias

Diagnosis	No of patients (n=90)	Percentage (%)
Bacterial pneumonia	32	35.55
Malignancy	28	31.11
Tuberculosis	20	22.22
Fungal infection	06	6.66
Foreign bodies	04	4.44

**Table 2:** Organisms causing bacterial pneumonia

Organisms	Number (n=32)	Percentage (%)
*Pneumococci*	16	50
*Staphylococci*	10	31.25
*Klebsiella*	06	18.75

**Table 3:** Radiological presentation in study group

Radiological presentation	No (120)	%
Consolidation	50	41.66
Collapse	25	20.83
Parahilar mass	13	10.83
Consolidation with effusion	08	6.66
Cavity	08	6.66
Collapse with effusion	05	4.16

There is preponderance of disease in males as compared to females. In the present study, bacterial pneumonia were found in 20 males (62.5%) and 12 females (37.5%) malignancy were found in18 males (64.44%) and in 10 females (35.56%), tuberculosis were found in 12 males (60%) and eight females (40%), fungal infection in four males (66.66%) and two female (33.34%) while foreign body were found in equal number of male and female patients i.e. in two patients.

## DISCUSSION

Development of the flexible FOB and various accessory instrument that can be inserted via the working channel has extended bronchoscopic exploration to the lung periphery. The instrument permits acquisition of tissue biopsy specimen, selective mucosal brushing, and broncheoalvoelar washings. Bronchoscopy is currently the primary means for diagnosis pulmonary malignancies.[1]

We found that FOB was diagnostic in 75% (90 cases) of patients whereas in the study conducted by Fein am0 and colleague[2] the FOB was diagnostic in 12 out of 14 (86%) patients. The study population was less as compared to present study.

In present study bacterial pneumonia was found in 32 (35.55%) patients and it is the commonest etiology. *Streptococcus pneumoniae was* grown in 16 (50%), *Staphylococcus* in 10(31.25%) and *Klebsiella* in six (18.75 %) patients indicating that even commonest organisms clear very slowly and require aggressive work up to diagnose them. Jay and colleagues[3] found that *Streptococcus pneumoniae* clears very slowly and takes about eight to 10 weeks. Delayed clearance was also associated with age more than 75 years, patient with chronic obstructive airways disease (COPD) and in chronic alcoholics. In a study conducted by Macfarlane and colleagues,[4] only 59% of all patients with *Streptococcus pneumoniae* pneumonia had a normal chest film eight weeks after diagnosis. Staphylococcal infection is also common in present study. It is more common in elderly and debilitated patients. Resolution is slow and residual fibrosis is common. Gram negative enteric organism like *Klebsiella pneumonia* is often slow to resolve and it takes approximately three to five months leaving residual scarring and fibrosis.

An important concern for the clinician in the timing of evaluation of various lung conditions which were not resolved by appropriate antibiotics is underlying thoracic malignancy, which can manifest in several ways. In the present study lung malignancy was found in 28 patients (31.11%) Johnson JL et al,[5] have reported various type of lungs malignancy in up to 11% patients. Bronchioloalveolar cell carcinoma is a sub type of adenocarcinoma that can be discerned as a lung mass or nodule or as a pulmonary infiltrate. Endobronchial mass lesions are not usually present at bronchoscopy in these patients; however, bronchial brushings, bronchioalveolar lavage and trans-bronchial biopsy are useful in making the diagnosis. A study by Feinsilver SH *et al*.[2] found adenocarcinoma and broncheoalvoelar carcinoma in 11% of patients at bronchoscopy, which has a low yield as compared to the present study. However, these might be due to less number of patients. Smokers over the age of 40 years and other patients at high risk for lung cancer who have recurrent or non resolving pneumonia should undergo bronchoscopy to detect endobronchial malignancy and post operative pneumonia.

In this study, tuberculosis was found in 20(22.22%) patients. In a retrospective review of patients over a six-year period, Baughman *et al*.[6] observed that bronchoscopy with BAL was useful in the diagnosis of pulmonary tuberculosis. In their study there were 30 patients whose pre-bronchoscopy expectorated sputum specimens were negative for AFB. Of these, bronchoscopy specimen were smear positive in 26 (87%). This is very high as compared to our study.

Kenedy *et al*.[7] had further observed that early diagnosis of sputum smear negative pulmonary tuberculosis was possible in 38% patients if different bronchoscopy procedures such as transbronchial biopsy and post bronchoscopy sputum, in addition to BAL, were studied. Panda *et al*.[8] reported that immediate diagnosis was possible in 35% of patients using transbronchial biopy and bronchoscopic lavage. But in all the above studies there were radiologicaly suspected cases of pulmonary tuberculosis and different bronchoscopic procedures are employed instead of single procedure during bronchoscopy.

Fungal pneumonia is also one of the causes of non resolving pneumonia. In our study, we found fungal pneumonia in six patients (6.66%). All the patients with fungal pneumonia were diabetic and had multiple cavitary lesions on chest radiography. The infection due to Candida species has become more frequent in immunocompromised patients like HIV, diabetes mellitus, patient on immunosuppressive drug and corticosteroids. The study conducted by Kyprianou A *et al*.[9] found fungal infection in 14% of the patients, which is quite high as compared to the present study. Other fungal infections like *Aspergillus and Cryptococcus* are also common in patients having depressed immunity.

Foreign body in major bronchi should be considered when pulmonary infiltrates do not clear. Retained foreign bodies occur more often in patients with impaired cough and protective airway reflexes and in intoxicated patients. Not all foreign bodies are radio-opaque and bronchoscopy should be considered in the appreciated setting even if a foreign body is not visible on chest X-ray. In our study we are found foreign bodies in 4 (4.44%) patients. The right lower lobe was the most common site for lodgment of the foreign bodies. In our study the commonest foreign body was beetle nut in all adult patients.

We conclude that FOB is useful in finding specific etiology of various lung conditions not responding to routine treatment. However, if the identifiable risk factor is not evident on FOB, further diagnostic testing with a thoracic Computerized Tomography scan and other studies should be considered.
